# Conduct Disorder-Related Hospitalization and Substance Use Disorders in American Teens

**DOI:** 10.3390/bs9070073

**Published:** 2019-07-05

**Authors:** Anum Masroor, Rikinkumar S. Patel, Narmada N. Bhimanadham, Sanjeetha Raveendran, Naveed Ahmad, Uwandu Queeneth, Amaya Pankaj, Zeeshan Mansuri

**Affiliations:** 1 Department of Psychiatry, Khyber Medical College, Khyber Pakhtunkhwa 25120, Pakistan; 2 Department of Psychiatry, Griffin Memorial Hospital, Norman, OK 73071, USA; 3 Department of Public Administration, Drake University, Des Moines, IA 50311, USA; 4 Centre for Addiction and Mental Health (CAMH), Toronto, ON M6J 1H4, Canada; 5 Department of Psychiatry, University of Texas, Houston, TX 77021, USA; 6 Department of Psychiatry, Maastricht University, 4–6, 6211 LK Maastricht, The Netherlands; 7 Jawaharlal Institute of Postgraduate Medical Education and Research (JIPMER), Puducherry 605006, India; 8 Department of Psychiatry, Texas Tech University Health Science Center, Midland, TX 79701, USA

**Keywords:** conduct disorder, adolescents, hospitalization, substance use disorder, cannabis abuse, opioid abuse, alcohol abuse

## Abstract

Objective: Our study aimed to compare the demographic characteristics of conduct disorder (CD) inpatients versus other psychiatric inpatients in children and adolescents, and assess the association between conduct disorder patients and the spectrum of substance use disorders (SUD). Methods: We included 800,614 psychiatric adolescent (12–18 years) inpatients, and this included 8885 inpatients (1.1%) primarily for conduct disorder in the Nationwide Inpatient Sample (2010–2014). ICD-9 codes were used to detect SUD, and a logistic regression model was used to evaluate the odds ratio (OR) for SUD in conduct disorder inpatients. Results: A higher proportion of conduct disorder inpatients were of 12–15 years of age (62.6%), male (64.4%), and White (45.7%). The lower median household income was correlated with a higher prevalence of conduct disorder (36.4%). Among SUD, cannabis use (23.7%) was most prevalent in conduct disorder inpatients followed by tobacco and alcohol use (10.1% each). Conduct disorder inpatients have 1.7-fold higher odds (95% confidence interval (CI) 1.52–1.82) for alcohol use and 1.4-fold higher odds (95% CI 1.31–1.49) for cannabis use compared to the non-conduct disorder inpatients. Cannabis use was seen significantly in adolescents (49.1%, 12–15 years), male (75.6%), and African Americans (45.6%). Conclusion: Conduct disorder inpatients have a higher risk of comorbid SUD compared to other psychiatric illnesses. The most common substance to be abused is cannabis followed by tobacco and alcohol. Varying pattern of substance use was seen by demographics and these predictors may help the clinicians for early diagnosis and treatment to improve overall health-related quality of life.

## 1. Introduction

Conduct disorder (CD) is a severe condition characterized by hostile and sometimes physically violent behavior and a disregard for others [1]. Multifactorial conditions have contributed to the development of conduct disorder. Children and adolescents with conduct disorder have frontal lobe impairment which may have an underlying genetic basis, besides a dysfunctional and disorganized home environment [2]. The lifetime prevalence of conduct disorder in the US is estimated to be 9.5%, i.e., 12% among males and 7.1% among females [3].

Substance use disorder (SUD) is a disease that affects an individual’s brain and behavior and leads to an inability to control the use of illicit substances [4]. Alcohol and tobacco are the most commonly abused substances by adolescents, followed by cannabis [5]. As per the national survey in 2017, 17% of the 12 years and older age population (approximately 44 million) reported the use of an illicit substance or heavy alcohol use during the prior year and 8% (21.4 million) met diagnostic criteria for a SUD [6]. One class of behavioral problem consistently associated with early-onset of SUD is conduct disorder. Conduct disorder increases the risk for substance use initiation at age 15 with a greater relative risk for illicit substances it continues until age 18 and the likelihood of initiating cocaine, amphetamines, inhalants, and club drugs continues to be highly elevated by 21 [7]. These populations have the highest rates of SUD in both male and female subjects [8].

There are questions regarding the widely documented comorbidity of conduct disorder with other mental disorders [9]. Although it is clear that conduct disorder is associated with other disorders, little is known about the prevalence of these comorbid disorders; specifically, very little data is available regarding the likeliness of having substance/alcohol use disorders as a comorbidity with conduct disorder. Thus, the purpose of this study is to determine the demographic predictors and association of comorbidities in children, especially SUD hospitalized with a primary psychiatric diagnosis of conduct disorder.

### Literature Review

Conduct disorder is a behavioral problem involving violation of important rules, societal norms, and laws. The most important risk factors that predict conduct disorder include impulsiveness, low IQ and low school achievement, inadequate parental supervision, punitive or erratic parental discipline, cold parental attitude, child physical abuse, parental conflict, disrupted families, antisocial parents, large family size, low family income, antisocial peers, high delinquency rate schools, and high crime neighborhoods [10]. However, for many risk factors, it is not known whether they have causal effects. Cadoret et al. examined children who had a biological family history of antisocial personality disorder who were adopted into either stable or pathologic homes. They determined the highest incidence of aggression and conduct disorder occurred in children who had both the family history of antisocial behavior and were placed in disturbed adoptive homes [11]. Youth diagnosed with conduct disorder have a higher degree of distress and impairment in virtually all domains of living than youth with other mental disorders [12]. Many studies have demonstrated the long-term impact of conduct disorder as a developmental precursor of antisocial behavior and criminality [9,13,14,15]. Conduct disorder diagnosed in childhood acts as a strong predictor of many problems in adolescence and adulthood, including mental illness, substance abuse, legal problems, school drop-out, and academic issues and occupational problems [16,17]. In two longitudinal studies, children with comorbid conduct and depressive disorders had a higher risk of late-onset criminality and antisocial behavior compared to those with only emotional issues [18,19].

Moreover, studies in the past have shown that conduct problems are associated with an increased risk of other mental disorders [9]. Many children with a conduct disorder may have coexisting conditions such as attention deficit hyperactivity disorder (ADHD) (3–41%), depression (0–46%) and anxiety disorder (0–41%) [20]. Despite the significant impact of conduct disorder on society, the economy, and healthcare, no prior studies have been done to determine demographic differences in the inpatient population with conduct disorder compared to other psychiatric disorders in children and adolescent populations in the United States. Substance abuse and dependence tend to develop rapidly following first use, suggesting that a narrow window of opportunity exists to prevent substance disorders once drug use has begun. Among youth who met criteria for conduct disorder, 52% also met criteria for a substance use disorder [21]. Multiple national population surveys have found that about half of those who experience a mental illness during their lives will also experience a substance use disorder and vice versa [22,23]. Although there are fewer studies on comorbidity among youth, research suggests that adolescents with substance use disorders also have high rates of co-occurring mental illness; over 60% of adolescents in community-based SUD treatment programs also meet diagnostic criteria for another mental illness [24]. Although conduct disorder has been related to an earlier rate of SUD, whether the impact on conduct disorder-related hospitalizations varies by substance type has not been examined.

To our knowledge, this is the first nationwide study to (1) compare the demographic characteristics of conduct disorder inpatients versus other psychiatric inpatients in adolescents, (2) assess the odds of association between conduct disorder inpatients and spectrum of SUD, and (3) discern the demographic differences across various SUD in conduct disorder inpatients.

## 2. Methods

### 2.1. Data Source

A retrospective cross-sectional analysis on the Nationwide Inpatient Sample (NIS) data (2010 to 2014) the Healthcare Cost and Utilization Project (HCUP) [9] was conducted to study patterns in demographics and prevalence of SUD in conduct disorder adolescent inpatients. The NIS is the largest database from 4411 hospitals and covers 45 states in the United States [25]. We applied the discharge weight (DISCWT) variable from the NIS to manage the national representation of our study population [26]. To protect the privacy of patients, physicians, and hospitals, the data was de-identified [25]. So we were not required the Institutional Review Board permission to conduct this study.

### 2.2. Inclusion Criteria

We included adolescent inpatients (12 to 18 years’ age) with a principal psychiatric diagnosis (N = 800,614). The study population were further divided into conduct disorder (N = 8885) and non-conduct disorder (N = 791,729) hospitalizations by detecting the ICD-9 codes 312.00-312.03, 312.10-312.13, 312.20-312.23, 312.4, 312.8, 312.81, 312.82, 312.89, 312.9, 313.81, 314.1, 314.2, 314.8 or 314.9 in principal diagnoses field DX1 [10,11] in the NIS. 

### 2.3. Variables of Interest

The demographic variables included in this study were age, sex, race, and median household income for patient’s postal (ZIP) code. These details were extracted from the patient’s discharge records [26]. To measure the differences in SUD in inpatients, the following were included by detecting the ICD-9 codes in other diagnoses fields DX2 to DX25 [26]: tobacco, cannabis, opioid, cocaine, and amphetamine and alcohol use disorders.

### 2.4. Statistical Analyses

We used descriptive statistics to measure the differences in demographics and SUD between conduct disorder and non-conduct disorder inpatients. We used a binomial logistic regression model to evaluate the odds ratio (OR) for SUD in conduct disorder-related hospitalization and were adjusted for and age, sex, race, and median household income. We then used bivariate analysis and Pearson’s chi-square test to measure the differences in demographics by SUD in the conduct disorder inpatients. A two-tailed *P* value < 0.01 was used to determine the statistical significance of the test result. All statistical analyses were done using SPSS version 25 (IBM Corp., Armonk, NY, USA).

## 3. Results

Overall 800,614 adolescent inpatients for psychiatric illnesses were included in this study and conduct disorder was seen in 8885 inpatients (1.1%) as principal discharge diagnoses.

A higher proportion of conduct disorder inpatients were in 12–15 years’ age (62.6%), male (64.4%) and Whites (45.7%). Males had 2.5-fold higher odds (95% confidence interval (CI) 32.44–2.69) for conduct disorder hospitalization then females. Regardless of the higher prevalence of conduct disorder in Whites, we found that African American and Native American/Asian adolescents have 2.1 to 1.6-fold higher odds for hospitalization in the adjusted regression model (Table 1). The lower median household income (<25th percentile) was correlated with the higher prevalence of conduct disorder (36.4%) and it decreases as the household income increases.

Among all SUD, cannabis use disorder (23.7%) was most prevalent in conduct disorder inpatients followed by tobacco and alcohol use disorders (10.1% each). After adjusting for demographic confounders and other SUD, adolescents with conduct disorder have 1.7-fold higher odds (95% CI 1.52–1.82) for alcohol use disorder and 1.4-fold higher odds (95% CI 1.31–1.49) for cannabis use disorder compared to the non-conduct disorder inpatients. 

**Figure 1:**
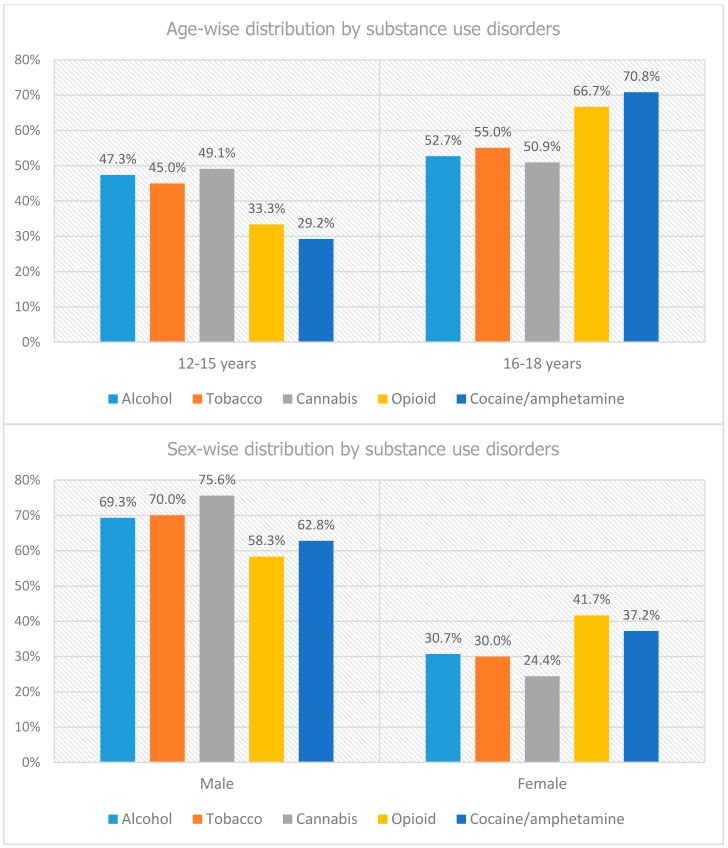

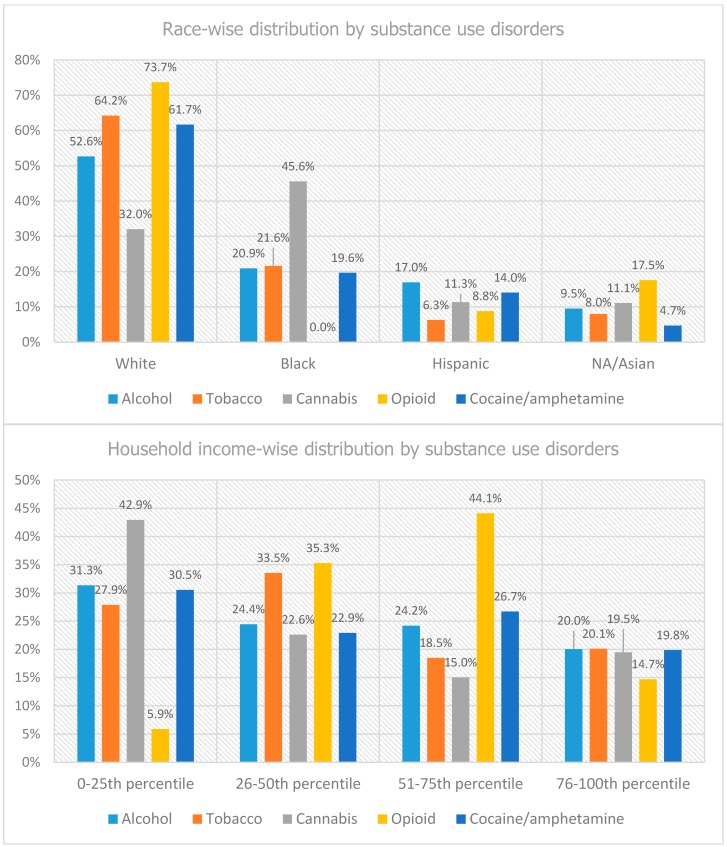
Demographic distribution of conduct disorder inpatients by substance use disorders.

We further categorized conduct disorder inpatients by SUD as presented in Figure 1. A higher prevalence of SUD (50.9% to 70.8%) was seen in conduct disorder inpatients between the ages of 16 to 18 years, namely cocaine or amphetamine (70.8%), opioid (66.7%), tobacco (55%), alcohol (52.7%) and cannabis (50.9%) use disorders. About three-fourths of the substances were abused by male, with 75.6% (highest) prevalence of cannabis use in male followed by tobacco (70%) and alcohol (69.3%) use disorders. Whereas, in females, opioid (41.7%), and cocaine or amphetamine (37.2%) use disorders were prevalent.

About half of African American adolescents abused cannabis (45.6%) followed by tobacco (24.6%). However, in Whites, substances majorly abused were opioid (73.7%), tobacco (64.2%), and cocaine or amphetamine (61.7%). Hispanics majorly abused alcohol (17%) and cocaine or amphetamine (14%). By socio-economic status (SES), adolescents with conduct disorder from low-income families (below 25th percentile) abused majorly cannabis (42.9%) and alcohol (31.3%), middle-income families (51st to 75th percentile) abused opioid (44.1%), and those from high-income families (above 75th percentile) equally abused alcohol, tobacco, cannabis, and cocaine/amphetamine as shown in Figure 1.

## 4. Discussion

Our analysis of nationwide inpatient data from the US hospitals is a first attempt to understand the demographic predictors for conduct disorder-related hospitalization in adolescents and association with spectrum of SUD. Our study findings of male preponderance (64.4%) in the conduct disorder inpatients corroborate the findings reported by McCabe KM et al. [27] who found that boys are more likely to have childhood-onset conduct disorder than girls because they had a more severe risk profile. Two previous studies have also concluded that boys are two to three times more likely to be diagnosed with conduct disorder than girls [28,29]. In our study, we found a higher proportion of conduct disorder in adolescents aged 12–15 years (62.6%) which correlates with the study by Patel et al. [28] which found that 55% of the children admitted to hospital for conduct disorder were adolescents (12 to 18 years).

Regardless of the higher prevalence of conduct disorder in Whites, we found that African Americans and Native American/Asian adolescents have higher odds for hospitalization and an inpatient study conducted by Patel et al. [28] that studied the demographic predictors for conduct disorder-related hospitalization also showed similar findings that African Americans were two times more likely to be admitted for conduct disorder than other race/ethnicities. Few other studies have shown that African American children are more likely to be diagnosed with conduct disorder with a lifetime prevalence of 8.1% [30,31]. Self-reported and observed aggressive and externalizing behaviors are more prevalent among Black youths [32,33], which suggests that CD diagnoses are likely to be higher as well [34,35]. Two possible reasons have been suggested as explanations for this apparent disproportional involvement of Black youth in aggressive activities (other than bias in reporting of criminal involvement). One is the fact that Blacks are more likely than Whites to live in lower SES environments [36,37], which are characterized by greater exposure to aggressive models [38], and higher rates of CD [39,40]. A second reason is a fact that Black youths are exposed to an additional source of stress that seldom affects White youths, and that is racial discrimination [41]. This stress has been linked with both internalizing and externalizing behaviors in adolescents [42]. More generally, stress from discrimination has often been suggested as a central factor contributing to the pronounced disparity in health status that exists between Blacks and Whites in the US [43,44,45]. Also, children from low-income families have serious concerns in terms of conduct and attention functioning [28,29]. As per our study findings, lower median household income (<25th percentile) was associated with the higher prevalence of conduct disorder hospitalization (36.4%) and it decreases as the household income increases. D’Onofrio et al. [46] concluded that there is a causal association between family income and conduct disorder, and low family income is a critical risk factor for the development of early-onset conduct disorder. 

Disney et al. [8] studied the relationships between ADHD, conduct disorder, and gender to SUD in a large population-based sample of adolescent twins. In this study, they concluded that a diagnosis of conduct disorder is an important predictor of SUD especially for cannabis, alcohol and tobacco abuse [8]. These findings coincide with our study that among all SUD, cannabis use disorder (23.7%) was most prevalent in conduct disorder inpatients followed by tobacco and alcohol use disorders (10.1% each). A study by Henry B et al. [47] shows that a strong association between conduct disorder and SUD emerged at age 15 for both males and females. Findings of Morse et al. [48] indicated that depressive symptoms mediated the relation between cannabis use and conduct disorder diagnosis.

Nearly 42% of the men diagnosed with conduct disorder met the diagnostic criteria for alcohol dependence [49]. In addition, nearly 23% percent of the women diagnosed with conduct disorder met the diagnostic criteria for alcohol dependence, high comorbidities exist between alcohol abuse/dependence and conduct disorder, particularly for the adolescent-onset subtype of conduct disorder [49]. There also exists an increased risk for a young person diagnosed with conduct disorder to develop an alcohol use disorder if one or both parents also had alcohol abuse [49].

A past history of conduct disorder increases the chances of substance use and abuse at age 16 [50]. In a study by Hopfer et al. [7] conduct disorder was associated with elevated adjusted hazards for initiation of all substances, with comparatively greater hazard ratios at age 15. By age 18, the adjusted hazard ratios remained significant except for alcohol. At age 21, the adjusted hazard ratios were significant only for cocaine, amphetamines, inhalants and club drugs [7]. A similar pattern was seen in our study for ages 16 to 18 years’ cocaine/amphetamine use disorders were prevalent followed by opioid, tobacco, alcohol, and cannabis whereas cannabis-use disorder was seen majorly in adolescents 12 to 15 years. Pacek et al. [51] showed that cannabis-use disorder was greatest among African Americans while cocaine-use disorder was more prevalent in the Whites, which is similar to our findings, as about half of African American adolescents abused cannabis (45.6%). However, in Whites, substances significantly abused were opioids (73.7%), tobacco (64.2%), and cocaine/amphetamine (61.7%). Another study states that among cannabis users, the odds of cannabis abuse and dependence were greater among African American, Native-Americans, and Hispanics than Whites [52].

A study by Patrick et al. [53] found that smoking in young adulthood was associated with lower childhood family socioeconomic status, while alcohol use and cannabis use in young adulthood was associated with higher childhood family socioeconomic status. By contrast, our study findings showed that adolescents from low-income families (below 25th percentile) abused majorly cannabis (42.9%) and alcohol (31.3%), middle-income families (51st–75th percentile) abused opioid (44.1%), and those from high-income families (above 75th percentile) equally abused alcohol, tobacco, cannabis, and cocaine/amphetamine. This variation could be due to the fact that we took into account only household income while the study by Patrick et al. [53] included education, wealth and income altogether. We focused on the demographic characteristics of adolescents with conduct disorder by SUD in order to delineate the predictors for the rising risk of SUD in these at-risk populations. Fergusson and Horwood [54] found that after controlling on a wide range of predisposing factors, including gender, family socioeconomic status, IQ, and childhood conduct disorder, a direct association remained between early marijuana use and poorer educational attainment and unemployment. Because adequate education is needed for successful functioning in a variety of adult roles, especially employment, and studies have shown that early marijuana use may lead to poor education [55] early marijuana use’s association with later poor outcomes may be an indirect one through education. There is mounting evidence that gender plays such an essential role in drug use. Men and women have different risk factors, opportunities to use, and rates of use [56,57]. Men and women also experience different social issues when it comes to drug use. For example, though substance use, in general, is stigmatized, women who use drugs encounter more social disapproval than men [58] a study [59] concluded that drugs are more likely to affect a man’s job or career path, whereas women are more likely to experience family problems, as evidenced by, for example, higher rates of divorce in substance-using women than men [60]. This selection and the consequences of early drug use may vary for males and females. A study [61] found that family factors influence the onset of drug use, especially among females.

Interestingly, for the illegal substances of cannabis, cocaine, and heroin, we found a consistent association with family factors for females only and no influence of family factors for males. Social implications of substance use include employment, marriage, and parenting. Also, success in employment and family relations have been linked to better long-term health, and high school graduation is a potential target for drug intervention programs [62].

Similarly, programs that focus on preventing or reducing teen substance use may increase the likelihood of high school graduation, diminish adult drug use, and have benefits about the transition to adult roles. Therefore, it seems necessary that those who design school dropout programs and drug prevention programs consider focusing on all the risk factors. Wealthier individuals have easier access to substances, both because of their financial means and their social activities [63].

On the other hand, low-income individuals have higher levels of stress, which leads to higher rates of drug and alcohol use. It is possible that the factors causing stress among wealthy and middle-class Americans—such as financial concerns, relationship conflicts, or parenting issues—are as influential and harmful as the factors that cause anxiety or depression among the poor. The lack of health insurance and financial resources is cited as a barrier to treatment in national surveys [63]. 

Research has indicated that there are several common pathways through which children and adolescents develop conduct disorder, each with different risk factors and each with different underlying developmental mechanisms leading to the child’s aggressive and antisocial behavior. Future research should consider whether other pathways could also explain the problems experienced by a significant number of youths with conduct disorder. It is important to note that this way of conceptualizing the development of conduct disorder has significant implications for how such research is conducted. Specifically, research should no longer focus simply on documenting what risk factors are associated with conduct disorder or which risk factors account for the most or the unique variance in measures of antisocial behavior, aggression, or delinquency. Such methods assume that conduct disorder is a unitary outcome. For future interventions, one key implication is the importance of prevention. As noted previously, the most aggressive youth and the youth most likely to continue their antisocial behavior into adulthood tend to show a childhood onset to their antisocial behavior [64].

Furthermore, several interventions have proven effective in treating early emerging conduct problems, with a significant decrease in their effectiveness in older children and adolescents [65]. Thus, intervening early in the developmental trajectory of childhood-onset conduct problems is an essential goal for preventing later severe aggression and antisocial behavior. Secondly, interventions need to be comprehensive and target multiple risk factors. Also, interventions need to be not only comprehensive but also individualized. That is, given that the causal processes leading to antisocial behavior appear to be different across subgroups of youths with conduct disorder [55]. Unfortunately, there is only very minimal research testing the utility of this matching of individuals with conduct disorder to different types of treatment depending on their unique characteristics, and this is a clear need for future research.

The key strength of this study is the national representation provided by the NIS dataset, with a uniform collection of data using ICD-9 diagnoses codes, and its large sample size of 800,614 adolescent inpatients. This study includes children with a psychiatric diagnosis of conduct disorder versus those without conduct disorder. The identification of patients using the ICD-9 diagnosis code may be affected by some external factors such as insurance and billing. Also, due to the cross-sectional nature of study we are not able to delineate a causal relationship between conduct disorder and SUD. Another major limitation is lack of information about the SUD patterns of consumption (e.g., weekly consumption, years of consumption, and polysubstance use). However, we included patients with a discharge diagnoses of SUD, and the clinical and non-clinical information in this dataset are coded independently of the individual practitioner and thus it is subjected to minimal reporting bias. Nevertheless, our study does have a few limitations as it is an administrative dataset, including lack of information of criminal history and other comorbidities including traumatic brain injury. Re-hospitalizations, which add to the total inpatient burden, was not accounted for in our study. However, despite these limitations, NIS is still an excellent population-based representation of disease associations with comorbidities.

## 5. Conclusions

White adolescents (aged 12–15 years) have a higher likelihood of conduct disorder-related hospitalization. These inpatients have a higher risk of comorbid SUD as compared to other psychiatric illnesses. The most common substance to be abused is cannabis followed by tobacco and alcohol abuse. Moreover, SUD is found to be more prevalent in patients of conduct disorder with the age of 16 to 18 years. With respect to gender, majority of the substances abused are seen in males with the highest prevalence of cannabis which is followed by tobacco and alcohol as compared to females in which opioids abuse is more common followed by cocaine and amphetamine. We also see a high prevalence of cannabis abuse/dependence in African American adolescents followed by tobacco as compared to opioids abuse in Whites which is followed by tobacco and cocaine and amphetamine. On the other hand, Hispanics majorly abused alcohol. It is not exactly known why some children develop conduct disorder. There are multiple factors involved in the disease process including traumatic events, social problems, and biological factors. To reduce the risk of conduct disorder, parents can learn positive parenting techniques. This can help to create a closer parent–child relationship, and also create a safe and stable home life for the child [64]. Early detection and intervention into negative family and social experiences may be helpful in disrupting the development of experiences that may lead to more disruptive and aggressive behaviors as seen in conduct disorder [64]. Specific attention should be given to the individuals who are at high risk of developing conduct disorder like youth with symptoms of impulsiveness, low IQ and low school achievement, poor parental supervision, child physical abuse, parental conflict, disrupted families, antisocial parents, low family income, antisocial peers and high crime neighborhoods [64]. Further studies should be done to highlight the growing issue of conduct disorder and its impact on comorbid SUD and the necessity to develop programs for early diagnosis and treatment to improve overall health-related quality of life.

## Figures and Tables

**Table 1:** Differences in conduct disorder (CD) and non-conduct disorder inpatients.

Variable	Conduct Disorder		Logistic Regression Model		
	No (%)	Yes (%)	OR	95% CI	*P* Value
Total inpatients	791729	8885	-	-	-
Age, in years					
12–15	49.1	62.6	1.95	1.85–2.05	<0.001
16–18	50.9	37.4	Reference		
Sex					
Male	42.0	64.4	2.56	2.44–2.69	<0.001
Female	58.0	35.6	Reference		
Race					
White	61.8	45.7	Reference		
African American	18.1	32.8	2.07	1.96–2.19	<0.001
Hispanic	12.5	12.3	1.12	1.04–1.21	0.005
Native American/Asian	7.6	9.2	1.57	1.44–1.71	<0.001
Median household income, in percentiles					
0–25th	27.0	36.4	2.01	1.86–2.17	<0.001
26th–50th	25.1	26.8	1.79	1.64–1.93	<0.001
51st–75th	24.4	22.7	1.53	1.41–1.66	<0.001
76th–100th	23.4	14.2	Reference		
Comorbid substance use disorder (SUD)					
None	-	-	Reference		
Alcohol	6.5	10.1	1.67	1.52–1.82	<0.001
Tobacco	10.5	10.1	0.88	0.81–0.96	0.004
Cannabis	15.5	23.7	1.40	1.31–1.49	<0.001
Opioid	2.2	1.6	0.72	0.59–0.88	0.001
Cocaine/amphetamine	2.0	2.8	1.29	1.11–1.51	0.001

OR: odds ratio; CI: confidence interval
